# Spread and scale of an electronic deprescribing software to improve health outcomes of older adults living in nursing homes: study protocol for a stepped wedge cluster randomized trial

**DOI:** 10.1186/s13063-021-05729-0

**Published:** 2021-11-02

**Authors:** Marc-Eric Nadeau, Justine L. Henry, Todd C. Lee, Émilie Bortolussi-Courval, Carole Goodine, Emily G. McDonald

**Affiliations:** 1 Centre for Innovation and Research in Aging, Fredericton, NB Canada; 2 Clinical Practice Assessment Unit, Department of Medicine, McGill University Health Centre, Montréal, QC Canada; 3 Department of Experimental Medicine, McGill University, Montréal, QC Canada; 4 Horizon Health Network, Fredericton, NB Canada; 5 Faculty of Health Professions, College of Pharmacy, Dalhousie University, Halifax, NS Canada; 6 Centre for Outcomes Research and Evaluation, 5252 De Maisonneuve Office 3E.03, Montréal, QC, Canada

**Keywords:** Polypharmacy, Deprescribing, Older adults, Long-term care, Medication use, Medication review, Adverse drug event, Nursing home, Prescription check-up

## Abstract

**Background:**

Medication overload or problematic polypharmacy is a major problem causing widespread harm, particularly to older adults. Taking multiple medications increases the risk of potentially inappropriate medications (PIMs), and residents in long-term care (LTC) are frequently prescribed 10 or more medications at once. One strategy to address this problem is for the physician and/or pharmacist to perform regular medication reviews; however, this process can be complicated and time-consuming. With a prescription review, medications may be decreased, changed, or stopped altogether. MedReviewRx is a software that runs an analysis using deprescribing rules to produce a report to guide medication reviews addressing medication overload for residents in LTC.

**Methods:**

This study will employ a mixed methods effectiveness-implementation hybrid type 2 study design. To measure effectiveness, a stepped wedge cluster randomized trial design is planned, which allows us to approximate a randomized clinical trial. Approximately 1000 residents living in LTC will be recruited from five facilities in New Brunswick. The study will begin with 3 months of baseline data on rates of deprescribing. Thereafter, every 3 months a new cluster will enter the intervention mode. The intervention consists of medication reviews augmented with the MedReviewRx software, which will be used by staff and clinicians in the facilities. The estimated study duration is 18 months and the main outcome will be the proportion of patients with one or more PIMs deprescribed (reduced/stopped or changed to a safer alternative) in the 90 days following a prescription review. The goal is to study the impact of MedReviewRx on medication overload among older adults living in LTC. In typical fashion of a stepped wedge cluster randomized trial, each cluster acts as an internal control (before and after) as well as a control for the other clusters (external control). Qualitative data collected will include resident/caregiver attitudes towards deprescribing and semi-structured interviews with staff working in the long-term care homes.

**Discussion:**

This study design addresses issues with seasonality and allows all clusters to participate in the intervention, which is an advantage when the intervention is related to quality improvement. This study will provide valuable information on PIM use, cost savings, and facilitators and challenges associated with medication reviews and deprescribing. This study represents an important step towards understanding and promoting tools to guide safe and rational reduction of PIM use among older adults.

**Trial registration:**

NCT04762303, Registered February 21, 2021.

## Administrative information

Note: the numbers in curly brackets in this protocol refer to SPIRIT checklist item numbers. The order of the items has been modified to group similar items (see http://www.equator-network.org/reporting-guidelines/spirit-2013-statement-defining-standard-protocol-items-for-clinical-trials/).

**Table:** 

**Title {1}**	Spread and scale of an electronic deprescribing software to improve health outcomes of older adults living in nursing homes: study protocol for a stepped wedge cluster randomized trial
**Trial registration {2a and 2b}.**	NCT04762303, Registered February 21, 2021 https://clinicaltrials.gov/show/NCT04762303
**Protocol version {3}**	Protocol version 1.0 of April 28, 2021.
**Funding {4}**	This research was financially supported by the Healthy Seniors Pilot Project, which is a $75 million three-year agreement between the Government of New Brunswick and the Public Health Agency of Canada, jointly led by the Government of New Brunswick’s Department of Social Development and the Department of Health through the Aging Secretariat.
**Author details {5a}**	Marc-Eric Nadeau, B.A.; Research Coordinator, Centre for Innovation and Research in Aging; MNadeau@ycc-cira.ca Justine L. Henry, BSc., MSc.Kin; Director of Research, Centre for Innovation and Research in Aging; JHenry@ycc-cira.ca Todd C. Lee MD MPH; Associate Professor of Medicine; Department of Medicine; McGill University Health Centre, Montreal QC, Canada; todd.lee@mcgill.ca Émilie Bortolussi-Courval; CPN and Doctoral Student; Department of Experimental Medicine, McGill University, Montréal, QC, Canada; emilie.bortolussi-courval@mail.mcgill.ca Carole Goodine, RPh, BSc (Pharm), ACPR, Pharm D; Clinical Pharmacy Manager, Fredericton Area, Horizon Health Network; Adjunct Professor, Faculty of Health Professions, College of Pharmacy, Dalhousie University. Carole.goodine@horizonnb.ca Emily G. McDonald, MDCM MSc FRCPC; Associate Professor of Medicine; Department of Medicine; McGill University Health Centre, Montreal QC, Canada; Director- Clinical Practice Assessment Unit, emily.mcdonald@mcgill.ca
**Name and contact information for the trial sponsor {5b}**	The Centre for Innovation and Research in Aging 100 Sunset Drive, Fredericton, New Brunswick E3A 1A3, Canada 1-506-444-3880 ext. 2560 MNadeau@ycc-cira.ca
**Role of sponsor {5c}**	The funding source has no role in the study design; data collection, management, and analysis; interpretation of the data; writing the manuscript; or the decision to submit the manuscript for publication. The content is solely the responsibility of the authors and does not necessarily represent the official views of the funding sources. MedSafer was funded by the Centre for Ageing and Brain Health Innovation (CABHI), The Canadian Frailty Network and the Canadian Institutes for Health Research. Initial development of the Polypharmacy App developed (designed) for YCC was funded by CABHI, the New Brunswick Health Research Foundation, AGE-WELL and the New Brunswick Innovation Foundation.

## Background and rationale (6a)

As highlighted by the Working Group on Medication Overload [1], problematic polypharmacy is an overwhelming problem for older adults. Nearly two thirds of community-dwelling adults are taking five or more medications [2] and while most medications are prescribed to help people live longer, healthier lives, the more complex a medication regimen is, the more dangerous it becomes. With each added drug, the risk of an adverse drug event (ADE) increases [3], as well as the worsening of geriatric syndromes [4]. A growing body of evidence suggests that patients and physicians may not recognize ADEs and additional new medications may be prescribed to treat an existing ADE [5]. This so-called prescribing cascade further increases polypharmacy and the risk of ADEs. Aging itself is a risk factor for ADEs as changes in organ function, body weight, fat distribution, cognition, balance, function, and drug receptor sensitivity all affect medication metabolism and side effects [6]. Experts in geriatrics have published guidelines and lists of medications to be avoided or used cautiously in older adults [7–9]. These medications are often referred to as potentially inappropriate medications or PIMs. PIMs are medications associated with a high risk of ADEs when administered to older adults and may be of uncertain or no clinical benefit [10]. Continued use of PIMs in later life further increases the risk of ADEs associated with polypharmacy in older adults. Furthermore, as highlighted in a 2018 systematic review, there is a significant association between an increased number of medications and frailty, which is associated with an increased risk of adverse events in older adults [11].

Older adults living in nursing homes are at a particularly high risk of polypharmacy and ADEs as they are frail and often take ten or more medications [2]. Some of these medications increase the risk of falls, fractures, kidney injury, bleeding, and death. Costs to the healthcare system from polypharmacy are very high and stem from emergency department visits, hospital admissions, and direct and indirect drug costs. As such, strategies to address polypharmacy are needed. One approach is the process of deprescribing, whereby a prescriber or pharmacist establishes the full list of medications a patient is taking and contextualizes them for the individual patient [12]. Based on the medical history, a measure of frailty, and the patient and/or caregiver’s values or preferences, safer classes of medications are selected, doses may be decreased, and medication may be discontinued [12]. This process can be thought of as a “prescription check-up.”

While deprescribing may be effective for stopping PIMs, manually reviewing all drugs and cross referencing them with patient conditions and lists of inappropriate drugs in older adults requires an expert command of the literature and is time-consuming. In many Canadian provinces, nursing home medication reviews are mandatory; however, there are no consistent guidelines for the reviews and therefore existing processes often result in medications being re-prescribed without documentation of a clear rationale or reassessment. Studies in acute care hospitals have demonstrated that electronic decision support can facilitate the process of deprescribing and augment the proportion of patients with one or more PIM (potentially inappropriate or problematic) medications stopped upon discharge from the hospital [13]. More studies looking at electronic tools for deprescribing in nursing homes are needed to determine if an investment on the part of the government into a software that supports deprescribing is warranted.

An electronic decision support tool for deprescribing called MedSafer has been studied in the Canadian acute care context. MedSafer augmented the proportion of patients who were discharged from the hospital with one or more PIMs deprescribed as compared to usual care [13]. New Brunswick (NB) has the highest proportion of older adults compared to other provinces in Canada. In NB, 20.8% of the population is over 65 years of age and this is expected to increase to 31.3% by the year 2038 [14]. Strategies to promote regular prescription check-up are needed to reduce polypharmacy in the setting of LTC. For this study, MedSafer researchers partnered with the Centre for Innovation and Research in Aging (CIRA) at York Care Centre (YCC) to improve a previously developed web-based application (polypharmacy app) that allowed prescribers in New Brunswick Nursing Homes (NBNHs) to visualize MedSafer deprescribing reports on a desktop computer or tablet. The polypharmacy app, called MedReviewRx, will be implemented in a number of NBNHs.

### Research questions

1. What is the impact of the MedReviewRx App on deprescribing PIMs in NBNHs (effectiveness)?
2. Is the MedReviewRx App developed at YCC, in Fredericton NB, feasible (easy to use), does it provide useful information, and does it reduce the time needed to conduct a medication review in other NBNHs?
3. How often do nursing home health care providers log into the MedReviewRx App, and how often does this action result in a prescription check-up (review and deemed appropriate or review and medication change)?
4. What are the challenges to implementing the MedReviewRx App in other NBNHs and what improvements are required for widespread use of the MedReviewRx App?

### Objectives [7]

To embed software enabled deprescribing in five NBNHs using MedReviewRx, an App containing MedSafer recommendations, in order to study the effectiveness at deprescribing PIMs, measured at the individual resident level.

To test the feasibility (ease of use) of implementing electronic deprescribing in LTC homes measured at the individual prescriber level.

### Trial design [8]

This is a hybrid type 2 effectiveness-implementation design for quality improvement research that will make use of mixed methods of evaluation. Implementation consists of using MedReviewRx. Effectiveness analysis consists of measuring the impact of the MedReviewRx on PIMs. Exploratory surveys (the mixed methods component of the study) which look at qualitative and quantitative user feedback and patient and caregiver attitudes about deprescribing will be used to explain study findings. Informal feedback, surveys and semi-structured interviews will be used to examine user experience with MedReviewRx. The Revised Patient Attitude Towards Deprescribing (rPATD) questionnaire “older adult” version for the patients and a “caregiver” version for family members/substitute decision makers will be used to describe patient and family member attitudes about deprescribing medications.

### Intervention description {11a}

Estimated study duration is 18 months, and the effectiveness component of the study will use a deployment strategy which approximates a stepped wedge cluster randomized trial (SW-CRT). This design has many advantages and is considered the most robust type of study design for pragmatic quality improvement interventions. Each cluster will contribute a control period. Subsequently, at each of 3 pre-defined time periods, one cluster will be randomly assigned to transition from control to intervention. In this way, all clusters act as an internal control (before and after) as well as a contemporaneous control for the other clusters (external control). The study is composed of three clusters and each cluster contains the following number of beds: Cluster A 218, Cluster B 314, and Cluster C 272.

InterRAI Long-Term Care Facilities (LTCF) assessments are standardized for nursing home residents. These assessments are used to help identify health issues among residents, develop care plans, and monitor changes in the health of LTC residents. InterRAI LTCF assessments are conducted quarterly for every person living in a nursing home in New Brunswick. These assessments contain sociodemographic information, medical diagnoses, information on medications used by the resident, and variables which describe cognitive and physical function. The interRAI LTCF assessment form is completed by gathering input from the care team and responses are inputted electronically into Momentum Healthware (Momentum) software by nurses or interRAI coordinators.

MedSafer is a computer program which cross-references medical conditions, ICD-10 diagnosis codes, and medication drug identification numbers (DINs) to generate a deprescribing opportunities report that can be used by the prescriber as a roadmap for a comprehensive prescription check-up.

**Fig. 1:**
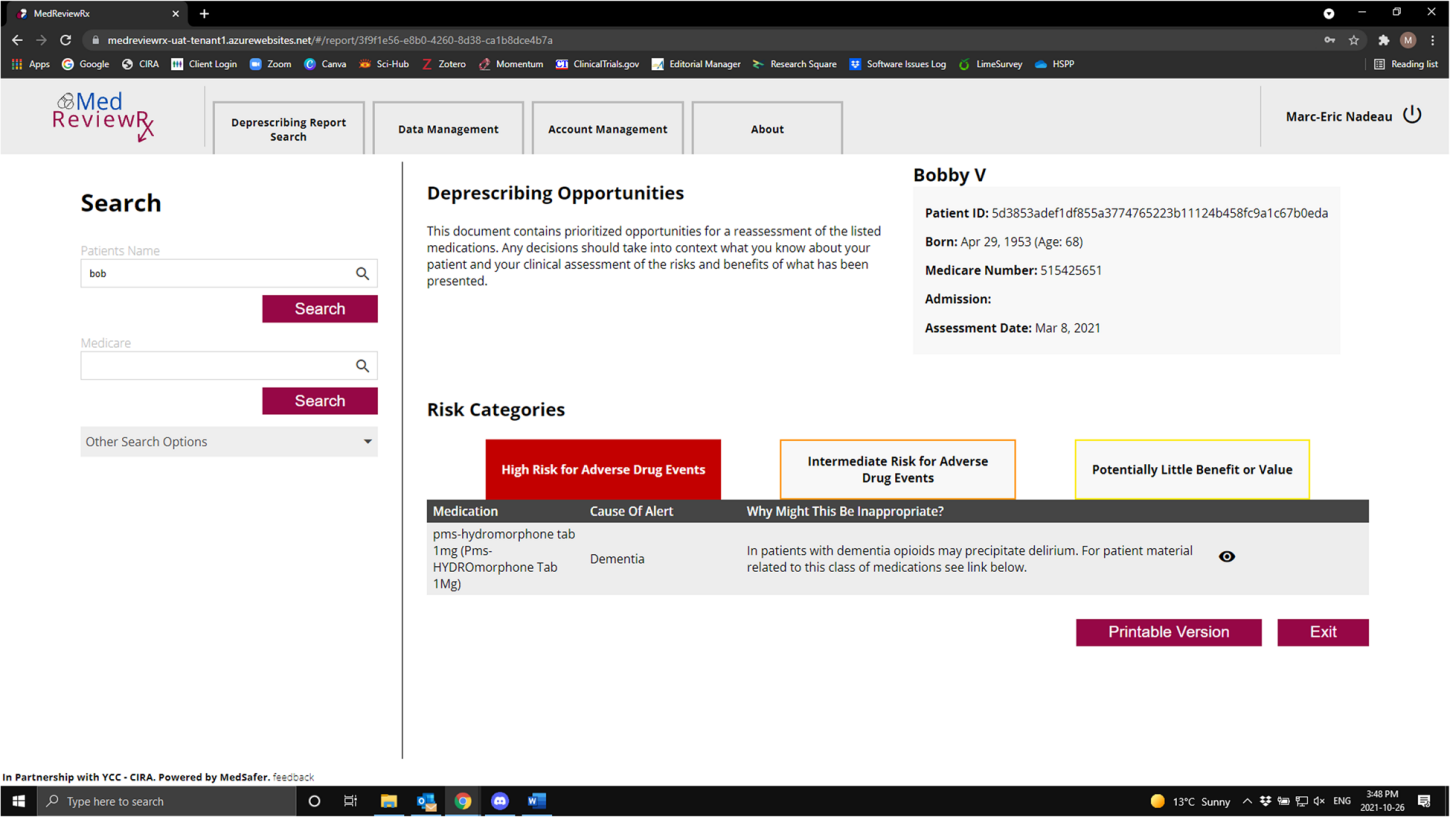
Sample screenshot of MedReviewRx App

**Fig. 2:**
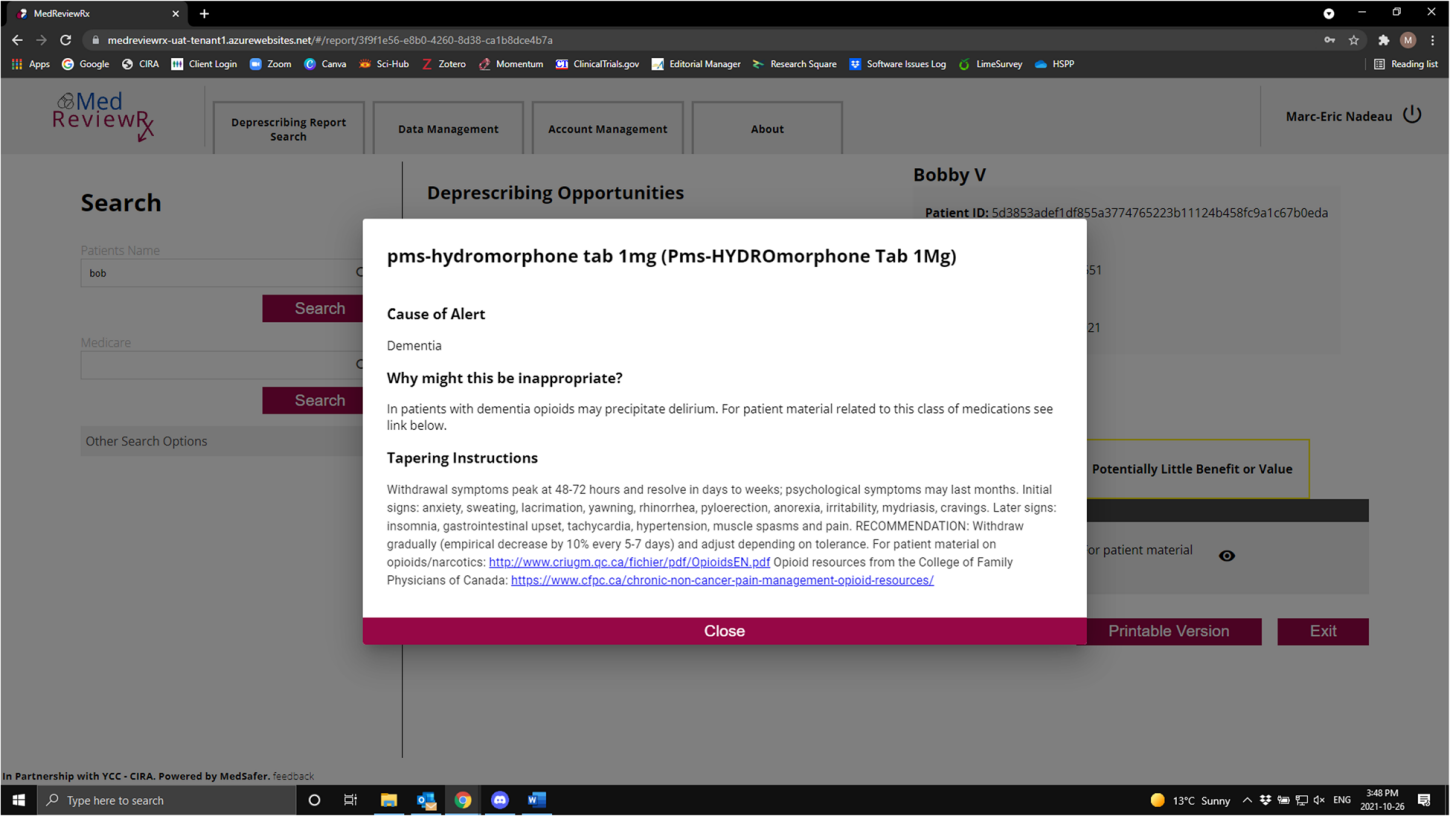
Sample screenshots of MedReviewRx App

The MedReviewRx system uses an application programming interface (API) to communicate with MedSafer. Patient assessments are downloaded from Momentum and uploaded into MedReviewRx at the nursing home. MedReviewRx removes identifiable patient information and assigns a unique identifier to each person’s data set, and anonymized data sets are encrypted and securely transmitted to MedSafer for analysis. MedSafer analysis is securely transmitted back to MedReviewRx where it is linked to the appropriate patient file and is available to be viewed by nursing home prescribers during their regular medication review. MedSafer deprescribing reports list medication(s) which have been flagged as potentially inappropriate, why the medication may be inappropriate, tapering instructions and links to patient and family materials if available. Figures 1 and 2 provide sample screenshots of the application.

### Criteria for discontinuing or modifying allocated interventions {11b}

There is no planned interim analysis as the intervention is considered safe and best practice. The study will be analyzed according to the intention to treat principle.

### Strategies to improve adherence to interventions {11c}

We will provide training for the software users, along with memos and email remainders about the study during the intervention phase. Ultimately, the decision to use the software will up to the individual prescriber as this is a pragmatic intervention to study the real-world effectiveness of making the software available for use in the LTC homes.

### Relevant concomitant care permitted or prohibited during the trial {11d}

All usual care received by the participant is allowed to continue during the trial.

### Provisions for post-trial care {30}

Participants in the trial will continue to receive usual care in the LTC home post-trial completion.

### Sample size {14}

Based on the Canadian Institute for Health Information’s report, titled “Drug Use Among Seniors in Canada, 2016”, we expect that about 70% will be taking one or more PIMs. This yields an average cluster size of 175 for patients with at least 1 PIM. Uptake of deprescribing using the MedSafer report in acute care studies ranges from 8 to 25% [13] over usual care. Conservatively, we think we can increase the proportion of patients with 1 or more PIM deprescribed from a baseline rate of 10 to 20% (absolute increase of 10% or a number needed to treat of 10). Using the Shiny CRT calculator [15], we estimate that we will have at least 72% power to detect this difference under assumptions of cluster autocorrelation of 0.8 and a within period intra-cluster correlation (ICC) 0.05. This increases to 96% power if the ICC is 0.02.

### Recruitment {15}

Nursing homes may experience 20 to 30% patient turnover each year due to deaths; therefore, the total number of nursing home residents who have at least one interRAI LTCF assessment during the study period is approximately 1000. For the purposes of analysis, only the initial closed cohort will be included in the study. New residents will not be analyzed in the current study. All homes have agreed to participate in the study and an individual waiver of consent has been granted by the Horizon Health Network Research Ethics Board.

### Sequence generation {16a}

Computer-generated random numbers were used in order to generate the allocation sequence of the clusters.

### Concealment mechanism {16b}

Study sites are not aware of which clusters they are assigned to and will only be made aware that they are entering into intervention mode approximately 4 weeks prior, to allow for training on the use of the software.

### Implementation {16c}

The principal investigator (EGM) of the study generated the allocation sequence. Participants are automatically enrolled in the study and assigned to the intervention when the targeted nursing homes agree to take part in the study.

### Who will be blinded {17a}

The study investigators and analyst will be blinded to the intervention status at the time of outcome adjudication. Prescribers cannot be blinded as the intervention is the use of a computer software.

### Procedure for unblinding if needed {17b}

There is no procedure for unblinding (does not apply to the trial design).

### Plans for assessment and collection of outcomes {18a}

Each study site will appoint a site contact to load information into MedReviewRx and to communicate with the study team. The nursing home site contact or designate(s) will be emailed a Nursing Home Site Assessment Questionnaire for completion prior to project implementation. The purpose of this questionnaire is to determine information about the nursing home including workflow, structure of medication reviews, physician, pharmacy, and nursing support. This information will be used to inform implementation and determine where MedReviewRx fits into the workflow. Study sites will also receive an implementation toolkit containing a user manual, instructions on how to use the Momentum extract report, a research study PowerPoint presentation, template for Staff/Crew Meeting “huddles”, sample announcement of study frequently asked questions, and a list of deprescribing resource websites. The CIRA research manager or designate will review the Nursing Home Site Assessment Questionnaire and Toolkit resources with the study site contact. If a nursing home site is not completing section N1 (list of all medications) of the interRAI LTCF assessment, a research assistant will be trained to enter this information for the study site.

The study will begin with a minimum 3-month control phase. During the control phase, MedReviewRx will not be accessible to healthcare professionals at the nursing homes. This serves to obtain baseline deprescribing levels for each nursing home. Every 3 months thereafter, a cluster of nursing homes will enter intervention mode.

During the control phase, nursing home residents and their families or substitute decision makers will be recruited to complete a survey to explore patient attitudes about deprescribing in this NB study population. The rPATD questionnaire “older adult” version will be provided to nursing home residents and the “caregiver” version will be provided to family members and substitute decision makers. Survey questions will be entered in Lime survey to promote electronic completion whenever possible. A recruitment poster will be displayed at study sites and a recruitment communication will be sent to family members and substitute decision makers identified in the resident’s admission package. Paper copies will also be available from the study site contact. Residents and family members who ask nursing home staff about the survey will have their contact information forwarded to the CIRA research manager to answer questions and obtain verbal consent to have a survey emailed to them. If email is not available, a research assistant will contact the participant to administer the survey over the phone. Study site contacts may also distribute paper copies of the surveys if requested and permitted by the study site.

During the control phase, health care providers and study site staff will be recruited to register for MedReviewRx. A recruitment communication will be sent by email and placed in study site mailboxes. Study recruitment will also be encouraged at interdisciplinary nursing home meetings such as Pharmacy and Therapeutics and Medical Advisory Committee meetings. User training will be provided by the study site contact or designate as determined by the site-specific implementation plan. Nursing home site contacts will participate in an interview to evaluate MedReviewRx implementation. Implementation interviews will be conducted within the first 3 months of the site intervention phase.

During the intervention phase, MedReviewRx will be made available to nursing homes with the understanding that it will be used to facilitate medication reviews and prescription check-ups. MedReviewRx (see Figs. 1 and 2 for screenshots of the App) provides clinicians with access to individualized and prioritized deprescribing information from MedSafer which:

A) Identifies PIMs.
B) Explains why the medication is potentially inappropriate.
C) Provides instructions on how to safely stop/taper the medication.
D) Links to patient informational brochures with non-pharmacologic interventions and rationale for deprescribing from the Canadian Deprescribing Network

Nursing homes will also have the option to print MedSafer deprescribing reports from MedReviewRx for clinicians who do not have computer access or who choose not to register to use MedReviewRx. If reports are printed, they will be placed in a binder for the prescriber to sign and date indicating that they have read the report. Prescribers and nursing home staff will be encouraged to write feedback directly on the report if they wish too. To assess the proportion of reports that were read, and feedback provided on the reports, signed reports will be kept in a locked cabinet for research assistants to collect. If visitor restrictions are in place, the study site contact will gather signed reports, place them in a sealed envelope(s), and send them to the CIRA research manager using a courier method that can be tracked and requires a sending signature and signature of receipt.

Clinicians will review deprescribing opportunities (electronically or via a paper report) and determine if medications can be tapered or stopped. Medication changes are discussed with the resident or substitute decision maker as part of usual care. This process will not change with the implementation of MedReviewRx. If the prescriber decides to alter a medication based on the deprescribing opportunities provided by MedSafer and their expert knowledge, the prescriber will do so in the same manner as they did prior to implementation of MedReviewRx, and discussion with the resident and/or substitute decision maker will occur according to the process in place at each nursing home.

During the intervention phase, an analysis of deprescribing opportunities will be conducted by MedSafer once every 3 months for each resident in the nursing home. Results will be stored in the MedReviewRx system to be accessed at any time.

Patients who die or are hospitalized will be censored from the study.

MedReviewRx has been designed to have Administrator accounts and Clinician accounts which restrict access according to user function. Study site contacts (or site designate) will load data into MedReviewRx using the Administrator function. Prescribers (physicians and nurse practitioners) and pharmacists will be provided with Clinician accounts. Clinicians will only access MedReviewRx records for patients under their care. MedReviewRx registration has been designed to restrict nursing home staff access to residents at their site only. Prescriber and pharmacist access will be restricted to study sites where they practice. MedReviewRx access will be audited by the CIRA research manager or designate.

User feedback on MedReviewRx and MedSafer deprescribing information will be solicited from prescribers, pharmacists, and nursing home staff throughout the study using surveys. Surveys may be completed via Lime Survey, on paper or by telephone. Three surveys will be distributed by email to registered MedReviewRx users as well as other study site prescribers, pharmacists, and nurses who may have access to printed reports from MedReviewRx. Paper copies of the surveys will also be available at the study site nursing units. A MedReviewRx user feedback survey will be distributed at the end of the first and third quarter of the intervention and an acceptability and feasibility survey will be distributed at the end of the study. Paper copies of completed surveys will be collected by the study site contact, placed in a sealed envelope(s), and sent to the CIRA research manager using a courier method that can be tracked and requires a sending signature and signature of receipt. Courier costs will be paid for by CIRA. MedReviewRx users will be provided with a study email which they may use to submit feedback and questions at any time throughout the study. Informal feedback received by email from users will be reviewed and actioned by the CIRA research manager or designate. Email feedback will be documented anonymously in an excel spreadsheet and reported to the study principal investigators within 1 week of receipt to determine if tasks need to be submitted to the technical team for software updates. Rapid cycle improvements in usability of the system and MedSafer output will be made based on survey results and informal feedback.

Informal feedback written on printed MedSafer reports will be documented in an excel spreadsheet and included in the analysis of qualitative feedback provided by survey and interview.

Frequency of MedReviewRx use will be measured electronically by counting the number of times a user accesses their account.

Overall experience with the research project will be evaluated using semi-structured interviews conducted at the end of the intervention period.

#### Sampling procedures

Convenience sampling was used to select nursing homes for the study. NBNH administrators and directors of nursing were contacted by email to determine interest in study participation.

Convenience sampling will also be used to recruit health care providers, older adults, and family members of older adults from study nursing homes. A recruitment poster for resident and family member surveys will be posted in study nursing homes and a communication will be sent to families and substitute decision makers by regular mail or email using the study site contact list. Health care providers and study site staff will be recruited to register for MedReviewRx by placing written recruitment notices in their nursing home site mailbox and/or by email. All pharmacists, nurses, nurse practitioners, and physicians providing care at the participating nursing home will receive recruitment emails for MedReviewRx feedback surveys. Members of healthcare teams at the study sites will be recruited to participate in interviews to explore overall experience with the research project.

#### Variables under investigation include:

A) Patient demographics (date of admission to LTC, age, sex, gender, nursing home identifier, medical, and psychiatric conditions documented in the interRAI LTCF assessment, and date of discharge from LTC if applicable).
B) Medication use data: absolute number of medications, number of PIMs, proportion of all medications that are PIMs, and type of deprescribing opportunities identified (PIMs that are high risk, intermediate risk, or of little added value).
C) Safety data: death, fracture, fall, use of restraints, transfer to hospital, change in functional status based on interRAI activities of daily living (ADL) scores. Episodes of delirium based on responses to questions in Section C of the interRAI LTCF assessment form (periodic disordered thinking or awareness and acute change in mental status from a person’s usual functioning).
D) D)Health care provider experience with MedReviewRx and MedSafer deprescribing information as described in the user feedback surveys and the acceptability and feasibility survey.
E) Nursing home evaluation of the implementation toolkit.
F) Patient and caregiver attitudes about deprescribing.

### Plans to promote participant retention and complete follow-up {18b}

Participants are automatically enrolled when targeted nursing homes agree to take part in the study.

### Data management {19}

Patient demographics and safety data is entered in the interRAI LTCF assessment software by nursing home staff as part of usual care. Medication information will be obtained from nursing home MARs and entered in section N1 of the interRAI LTCF assessment software by nursing home staff or a trained research assistant. Research assistants who enter medication data will receive training on how to enter medication information into Momentum LTCF assessment software. Patient demographics, medication use, and safety data will be extracted from the LTCF interRAI assessment software and loaded into MedReviewRx by the study site contact (or designate) at each nursing home.

### Confidentiality {27}

MedReviewRx will de-identify the data and transmit data sets to MedSafer. Multiple safeguards have been put in place to protect this data. Data sets do not contain patient-specific information. Age is transmitted rather than date of birth, and a unique identifier is assigned to each patient data set. MedSafer is unable to identify patients however the unique identifier allows MedReviewRx and MedSafer applications to anonymously communicate about specific patients through an application programming interface (API). Data sets will be analyzed by the MedSafer software program. Files containing one or more targeted PIM and associated triggering condition(s) will have deprescribing opportunities flagged and returned to MedReviewRx. MedReviewRx will link the unique identifier to the correct nursing home patient and translate the MedSafer analysis into an output for review by their health care team. A message will also be displayed by MedReviewRx if there are no deprescribing opportunities identified for a data set. MedReviewRx transmits and stores data using a secure web-based system which was designed by Missing Links Technology (MLT) under the supervision of the New Brunswick Community College (NBCC). The MedReviewRx system safeguards personal health information (PHI) that it collects, processes, and disseminates within trusted and authorized circles of care and ensures the confidentiality and privacy of the PHI. Each study site using MedReviewRx has site-specific policies and procedures with respect to privacy and confidentiality of PHI.

Data from User Feedback Surveys and Acceptability and Feasibility Surveys and survey responses collected from the rPATD questionnaires completed via Lime Survey will be extracted into excel sheet format. Survey data collected from paper surveys or telephone surveys will be entered into the excel database by a research assistant. Research assistants will be provided with templates for excel data entry and trained on how to complete the template. A minimum of 5% of the research assistant data entry will be audited for accuracy and completeness by the CIRA research manager or designate. Interview data will be recorded and transcribed verbatim by research assistants. Interview recordings will be deleted after transcripts have been validated. Study data (PIMs, survey responses, interview transcripts) will be stored in password-protected, members only secure cloud-based study sites hosted within Canada. Study data will only be accessible to the study research team and the CIRA research manager. On study closure, after final data analysis is completed, study data will be downloaded to a password-protected study folder on the YCC secure network and the cloud-based study site will be permanently deleted. Study data will be stored for 7 years and then destroyed in accordance with YCC/CIRA policies and procedures.

### Plans for collection, laboratory evaluation, and storage of biological specimens for genetic or molecular analysis in this trial/future use {33}

No biological specimens were collected as part of this trial.

Data analysis plan

This mixed methods study will employ both qualitative and quantitative data analysis.

### Statistical methods for primary and secondary outcomes {20a}

Analysis of the primary outcome will be conducted using the stepped wedge function in Stata v. 15 or a similar statistical program. The primary analysis will use a Generalized Liner Mixed Model with a logit link, treating time and intervention as fixed effects, and cluster as a random effect. This information has been added to the manuscript.

### Interim analyses {21b}

There is no planned interim analysis.

### Methods for additional analyses (e.g., subgroup analyses) {20b}

Subgroup analyses will be conducted using the stepped wedge function in Stata v.15 or a similar statistical program. We will also perform 2 sensitivity analyses that will include a completely fixed effects model as well as non-parametric permutation tests. This will address any concerns related to the low number of clusters.

### Methods in analysis to handle protocol non-adherence and any statistical methods to handle missing data {20c}

The study will be analyzed according to the intention to treat principle. All data that is collected is from the LTC inteRAI form and data entry is mandated by the province. We do not expect missing data.

### Plans to give access to the full protocol, participant-level data, and statistical code {31c}

The full protocol will be published online and anonymize participant-level data will be made available upon request to the principal investigator (EGM). Data will be made available for 1 year following the publication of the primary manuscript from the trial.

### Outcomes {12}

The co-primary outcomes of interest are:

1. The effectiveness of MedReviewRx for deprescribing based on the proportion of participants with one or more PIMs deprescribed, compared to usual care.
2. Feasibility of the intervention (see below for complete definition).

#### Impact on PIMs

The impact on PIMs will be determined by the proportion of nursing home residents who have one or more PIMs reduced or stopped after the treating physician receives a MedSafer report. This outcome will be measured by using unique identifiers to compare medication data from sequential interRAI data sets. PIMs identified in the baseline data set will be compared with the next data set (3 months later) transmitted with that unique identifier to determine if any of the PIMs has been stopped (no longer listed) or if a PIM dose has been reduced. This process will continue for the study duration. All statistical comparison will be performed via STAT v 15 (or equivalent) and the stepped wedge function will be used for the primary outcome (proportion of patients with one or more PIMs deprescribed over the study period).

At the end of the study, subgroup analysis will be conducted on the PIM data to explore PIM use based on sex, gender, and geographic location.

Co-primary outcome:

1. User experience with MedSafer reports and MedReviewRx.

#### User experience

User experience with MedSafer reports and MedReviewRx will be measured using survey responses as well as described through interview data and informal feedback received by the study team. Several outcomes will be studied: how satisfied the user is with the App, including how helpful they find it, the added knowledge from a MedSafer report for the prescriber, how likely the prescriber is to continue using the App once the study is over. Survey responses will be analyzed based on the following themes: value and benefits, scientific content, and user satisfaction. Interview data will be thematically analyzed [16].

Secondary outcomes to be examined at 90 days following each intervention cycle include:

A) Although we are not powered to show a reduction in other outcomes, we will explore any impact of the intervention on reduction of fractures, falls, use of restraints, transfer to hospital, change in functional status (based on interRAI activities of daily living (ADL) scores), episodes of delirium (based on responses to questions in Section C of the interRAI LTCF assessment form: periodic disordered thinking or awareness) and death.

#### Tertiary outcomes include:

A) Cost savings analysis related to cost saved from medications (actual price of the medication as well as dispensing fees) balanced with the cost of deployment of MedSafer including maintaining the program with updates and user support.
B) Patient and family attitudes about deprescribing will be reported using survey responses collected from the rPATD questionnaires.

#### Project evaluation

This project will be evaluated locally at each participating nursing home site to determine usability, impact on workflow, time commitment, and value added. Qualitative and quantitative user feedback as described above will be used to explain study findings.

Patient and family attitudes about deprescribing will be described descriptively using survey responses collected from the revised rPATD questionnaires.

An analysis will be conducted to determine strategies to inform widespread deployment, nursing home evaluation of the implementation toolkit, prescribing patterns based on geographic area and overall change in PIM use. System-level impact on cost savings and cost avoidance of adverse effects will be projected.

### Study setting [9]

A subset of nursing homes in New Brunswick have agreed to participate in the study. Study participants will include health care providers and nursing home staff at participating nursing homes, as well as the older adults who live in these nursing homes and their family members or substitute decision makers.

Our study represents a total 804 nursing home beds and will provide a sample of NBNH residents and health care providers in three urban centers representing both large and mid-size long-term care facilities who conduct business in English, as well as one long-term care facility being bilingual (English and French).

### Eligibility criteria [10]

#### Inclusion criteria

- Residents residing in one of the participating homes
- Aged 65 years of age or older
- Taking one or more PIMs (as identified by MedSafer)

#### Exclusion criteria for the secondary outcome of attitudes towards deprescribing

If both the patient and proxy are unable to complete the survey, the patient will be excluded from the survey component of the study.

### Participant timeline {13}

**Table:** 

	Study period						
	Allocation^**1**^	Post-allocation				Follow-up data	Close-out
Timepoint	***0***	***Months 1–3***	***Months 4–6***	***Months 7–9***	***Months 10–12***	***Months 13–15***	***Months 16–18***
**Enrolment**							
Eligibility screen	X						
Informed consent	X						
**Control (C) Intervention (I)**							
Cluster 1		C	I	I	I		
Cluster 2		C	C	I	I		
Cluster 3		C	C	C	I		
**Assessments**							
Demographic data		X	X	X	X	X	
Medication data		X	X	X	X	X	
Safety data		X	X	X	X	X	
Nursing Home Site Assessment Questionnaire		X					
RPATD^**2**^ Questionnaire: Older Adult Version		X					
RPATD^2^ Questionnaire : Caregiver Version		X					
User Feedback Survey			X		X		
Acceptability and Feasibility Survey						X	
Implementation Evaluation Interview			X	X	X		
End Of Study Feedback Interview					X		
**Data analysis**						X	X

^1^As this is a cluster randomized controlled trial, allocation occurs at the cluster level

^2^*RPATD* Revised Patient Attitude Towards Deprescribing

### Who will take informed consent? (26a)

A waiver of informed consent for participation in the deprescribing intervention aspect of the study as well as for secondary use of data was obtained from the research ethics board of the Horizon Health Network Research Ethics Board. Informed consent for participation in the survey component of the study will be obtained at the time of survey completion with a preamble prior to any survey completed. Residents who are unable to provide informed consent for surveys will have a surrogate decision maker approached when available. All residents regardless of capacity to consent are eligible for the quality improvement intervention due to a waiver obtained from the research ethics board.

Additional consent provisions for collection and use of participant data and biological specimens {26b}

Not applicable. No biological specimens will be collected as part of this trial.

### Explanation for the choice of comparators {6b}

Deprescribing using MedReviewRx during the intervention phase will be compared with usual care (deprescribing when not using MedReviewRx) in the control phase.

Prescribers (physicians and nurse practitioners) and pharmacists providing care at the study nursing homes will be eligible to use MedReviewRx to conduct prescription check-ups for older adults under their care. Nursing home staff (unit clerks, administrators, interRAI coordinators, and nurses) at the study site will be eligible to register to load LTCF assessment information into MedReviewRx and print MedSafer reports from the system for prescribers who do not use MedReviewRx. A designated contact person will be appointed by the Director of Nursing or designate at each nursing home. The study site contact will act as a liaison with the research study team and coordinate study site activities. Study site contacts will also complete a site assessment questionnaire at the beginning of the study and an interview within 3 months of initial MedReviewRx implementation. Prescribers, pharmacists, nurses, and staff at study nursing homes will be recruited to complete surveys to evaluate the MedReviewRx system and MedSafer report information. At the end of the study site contacts, nursing home staff, prescribers, pharmacists, and nurses will be recruited to participate in an interview to evaluate the program. Consent will be obtained for MedReviewRx users, interview, and survey participants.

Adults aged 65 years or older who live at participating nursing homes and have had a quarterly interRAI LTCF assessment completed are eligible for MedSafer analysis. A waiver of patient consent was requested and approved to load patient data into MedReviewRx. InterRAI LTCF assessments are completed on admission to a NBNH, and every 3 months thereafter as a standard of care. Assessment data is collected by NBNHs electronically using Momentum software. Momentum has written a customized data extract report for this project. Patient demographics, medical conditions, medication information, and safety monitoring data required for this study are extracted from the Momentum extract report and uploaded into MedReviewRx at the study site. MedReviewRx removes identifiable patient information and assigns a unique identifier to each person’s data set, and anonymized data sets are encrypted and securely transmitted to MedSafer for analysis. The ability to download secondary interRAI assessment data on medical conditions, medication lists, and safety data for each nursing home resident is essential to this research study. Without this information, it will not be possible to calculate the proportion of PIMs used at the study sites, evaluate the impact of MedReviewRx on deprescribing at each site, or assess study safety endpoints. A waiver of consent is unlikely to adversely affect the welfare of individuals to whom the information relates as the information is already collected as part of usual care. Study researchers have conducted an analysis of the MedReviewRx system and appropriate measures have been taken to protect the privacy of individuals and to safeguard personal health information. It is impracticable to seek consent from all individuals to whom the secondary information relates as new nursing home residents are admitted regularly when previous residents are discharged or deceased, interRAI data is downloaded in batches by assessment date, and study endpoints require information on all residents in each study site. In addition to safeguarding information, the research team will enter into data sharing agreements with each study site to obtain permission for secondary use of information for this research study.

Adults aged 65 years or older who live at a participating nursing home during the study, and their family members or substitute decision makers will be recruited to participate in a survey to explore their attitudes on medication use and deprescribing. Posters will be placed in study nursing homes; the study will be promoted verbally at resident and family council meetings and a recruitment communication will be sent by email or mail to family members identified as contacts and/or substitute decision makers in the resident’s admission documents. Consent will be obtained for survey completion.

#### Materials

1. Electronic documentation of medications and medical conditions in Momentum interRAI LTCF assessment software.
2. MedSafer software system for identifying PIMs.
3. A secure Cloud Service to store information. This will be obtained through a subscription from Canadian Web Hosting.
4. MedReviewRx, a system which extracts, de-identifies, and transmits data between study nursing homes and MedSafer and provides an interface for viewing MedSafer outputs on mobile devices and computers.
5. Nursing Home Assessment Questionnaire
6. Implementation Toolkit
7. User Feedback Survey
8. Acceptability and Feasibility Survey
9. rPATD questionnaire “older adult” version for the patients and a “caregiver” version for family members and substitute decision makers.
10. Recruitment materials

### Composition of the coordinating center and trial steering committee {5d}

The Centre for Innovation and Research in Aging (CIRA) is acting as the coordinating center. The Research Coordinator at CIRA, under its Executive Director, is in direct contact with all of the study sites participating in the trial in order to implement the intervention as intended. The trial steering committee comprises the Principal Investigators of the study (representing Horizon Health Network (HHN) and McGill University Health Centre (MUHC)) and a representative from CIRA. The trial steering committee meets online every 2 weeks. The data management team is being represented by the NB Institute for Research, Data and Training (NB-IRDT) and is providing the statistical analysis.

### Composition of the data monitoring committee, its role, and reporting structure {21a}

Data quality is being monitored by the above entities CIRA, HHN, MUHC, and NB-IRDT. A formal Data Safety Monitoring Committee was not felt to be necessary per the study team and as per the research ethics board. This study is a quality improvement intervention and is a study of process and implementation, with effectiveness and feasibility as a measure of the primary outcomes. Deprescribing has been studied extensively and is considered safe for older adults with polypharmacy living in long-term care homes. We do not seek to retest the safety of deprescribing but rather how best to implement the process using a software to facilitate report visualization.

### Adverse event reporting and harms {22}

We do not expect reports to contain errors. MedSafer has already been extensively tested on older adults with polypharmacy in the acute care setting (up to one third of whom were from the long-term care setting). If a clinician suspects that a report contains an erroneous recommendation, they are encouraged to email the study team for support. The study email is monitored daily during regular business hours.

### Frequency and plans for auditing trial conduct {23}

Each site will have a site initiation visit to go over the study protocols as well as a closing visit at the end of the study. Data is routinely collected data in the electronic health record. Feedback from the end users is being solicited through surveys and small group interviews.

### Plans for communicating important protocol amendments to relevant parties (e.g., trial participants, ethical committees) {25}

If any important protocol amendments take place, they will be communicated in writing, in an email format, to the relevant parties.

### Dissemination plans {31a}

Trial results will be made available through publication in a peer-reviewed indexed journal. Study results will be disseminated via newsletters to the study homes and pertinent results of the study will be communicated to New Brunswick health officials.

## Discussion

The goal of this project is to study the impact of MedReviewRx, an intervention designed to help address the issue of medication overload among older adults living in NBNHs. MedReviewRx allows health care providers (prescribers, pharmacists, and nurses) in nursing homes to access MedSafer analysis on a tablet or laptop. The intervention will be tailored and adapted to each participating nursing home’s existing administrative and management workflow. Our mixed methods study, employing both qualitative and quantitative data analysis, has been designed to measure several important outcomes. We will measure effectiveness by determining the proportion of patients with one or more PIMs deprescribed following each cycle of the intervention. Although we are not powered to examine if the intervention reduces individual rare outcomes, we will explore any impact on reduction in falls, fractures, use of restraints, transfers to hospital, change in functional status, episodes of delirium, and death. As a co-primary outcome, we will study the feasibility of the intervention as measured by user experience with MedSafer reports and MedReviewRx as part of the implementation process.

We will examine direct cost savings from deprescribing medications, and indirect costs saved from dispensing fees, balanced with the cost of deployment of MedSafer including maintaining the program with updates and user support. Moreover, we will measure patient and family attitudes about deprescribing using survey responses collected from the rPATD questionnaires.

In terms of our study design, we adopted a SW-CRT design. This study design addresses issues with seasonality and allows all clusters to participate in the intervention, which is an advantage when the intervention is related to quality improvement, and is appropriate for this study in several ways [17]. First, the SW-CRT design provides an opportunity for all participating nursing homes to receive the intervention at different time-points during the intervention period. This allows a staged implementation which helps when there are limited personnel to support the study (but from the research team and at the individual homes) while at the same time ensuring the quality of implementation. Third, the study design allows an estimation of the intervention effects using both a between- and within-cluster comparison. This unidirectional crossover design combines elements of before-after studies with cluster randomization and is an efficient design for answering the research question [18]. Moreover, stepped wedge trials can have a statistical advantage over parallel cluster randomized trials [19] [20]. The stepped wedge design is more efficient than a parallel group design as it requires a substantially smaller sample size.

We are using a cluster design to avoid contamination at the level of the home. The intervention involves a component of learning/teaching to the prescriber. If residents were randomized at the individual level within the same home, there is risk of contamination. Most homes have only a couple of prescribers. The intervention is directed at the level of the prescriber and so with time, they may learn that a certain medication is inappropriate, which risks contaminating participants who are randomized to the control. By randomizing at the level of a home, this minimizes this form of contamination.

Challenges in conducting the study are currently being faced. Firstly, the present pandemic era created by the novel COVID-19 has deprived our participating sites of their usual financial and human resources, which were allocated to battle the virus. Some of our homes even faced an outbreak of the virus among their residents, which put a halt on their research operations in order to fully mobilize their resources. Because every site is in its own unique situation, guidance and support from the study team will be tailored individually to each of those sites.

Our study has limitations. Our sample profile represents only a subset of New Brunswick nursing homes. Data from nursing homes in other provinces and other countries would provide a broader, diversified perspective to the current polypharmacy landscape. Our intervention may not apply to provinces and countries where medication reviews are not mandated, or in the absence of a unified electronic medical record for nursing home residents. Increased funding for a prolonged study period (lasting multiple years) would allow us to collect more data on the sustainability of the intervention. Finally, a larger sample size would increase our power to detect a difference in more rare outcomes such as preventable hospitalizations and death.

That said, the study has several advantages. For healthcare professionals, providing deprescribing reports will bring greater awareness to the risks of polypharmacy and PIMs. The convenience of easily accessing the reports online should also facilitate the process of deprescribing, rendering it more scalable. And while residents may not always be aware that their health care professional is using MedReviewRx to assess their medication regimen, they stand to benefit directly from the intervention through deprescribing medications that could be harmful, or of little added value. The result for the patient should be a decreased medication burden. Finally, from a health services perspective, we expect time savings for the pharmacy team by partly automating the medication review process.

In conclusion, this study will provide valuable information on PIM use as well as facilitators and challenges associated with medication reviews and deprescribing. We hope that this study will demonstrate that implementing MedReviewRx is feasible and acceptable to health care providers in NBNHs and that it results in a decreased PIMs and cost savings. This study is an important step towards understanding and promoting tools to guide safe and rational reduction in PIM use among older adults. We believe that MedReviewRx will be an efficient, user-friendly application that will be very useful to physicians, pharmacists, and nurses and will greatly facilitate the deprescribing process.

### Trial status

Protocol version 1.0 of April 28, 2021. Recruitment will begin in August 2021 and is expected to be completed by August 2022. Follow-up will be complete as of November 2022.

The study is nearing the beginning of the intervention phase at the submission of this manuscript. Data analysis has not yet started. Detailed statistical analysis plan will be complete prior to subject enrolment.

## Data Availability

Study protocol will be made available 1 year following the publication of the primary manuscript from the trial.

## Abbreviations

ADE: Adverse drug event
API: Application programming interface
PIM: Potentially inappropriate medications
LTC: Long-term care
LTCF: Long-Term Care Facilities
NB: New Brunswick
NBNHs: New Brunswick nursing homes
CIHI: Canadian Institute for Health Information
DINs: Drug identification numbers
CIRA: Centre for Innovation and Research in Aging
YCC: York Care Centre
MARs: Medication Administration Records
SW-CRT: Stepped wedge cluster randomized trial
CRTs: Cluster randomized trials
rPATD: Revised Patient Attitude Towards Deprescribing
MLT: Missing Link Technologies
NBCC: New Brunswick Community College
